# The perplexing role of immuno-oncology drugs in osteosarcoma

**DOI:** 10.1016/j.jbo.2021.100400

**Published:** 2021-10-22

**Authors:** Alannah Smrke, Yuen B. Tam, Peter M. Anderson, Robin L. Jones, Paul H. Huang

**Affiliations:** a BC Cancer, Vancouver, BC V5Z 4E6, Canada; b Sarcoma Unit, Royal Marsden Hospital, 203 Fulham Road, London SW36JJ, UK; c The Institute of Cancer Research, 15 Cotswold Road, Sutton SM2 5NG, UK; d Pediatric Hematology Oncology and Bone Marrow Transplantation, Cleveland Clinic R3 Main Campus, 9500 Euclid Avenue, Cleveland, OH 44195, USA

**Keywords:** Osteosarcoma, Systemic therapy, Immunotherapy, PDL1

## Abstract

Osteosarcoma is a rare, primary tumour of bone. Curative treatment consists of multi-agent chemotherapy and complete surgical resection. Despite the use of multi-agent chemotherapy, the risk of recurrence is high. Survival outcomes for patients with osteosarcoma have not changed since the 1980′s. Based on biologic rationale, there has been interest in adding immunotherapies to upfront curative intent chemotherapy, including mifamurtide (a macrophage activator) and interferon. However, results to date have been disappointing. In the metastatic setting, checkpoint inhibitors alone have not proven effective. Ongoing translational work is needed to further understand which patients may benefit from immune-oncology approaches with standard cytotoxic chemotherapy.

## Highlights

• Osteosarcoma outcomes have not improved since use of cytotoxic chemotherapy.
• Addition of macrophage activators and interferon have been disappointing.
• Combination therapies may be needed to exploit the role of the immune system.

## 1 Introduction

Primary sarcomas of bone are rare, aggressive neoplasms which often require intensive multimodality treatment. Osteosarcoma, Ewing Sarcoma and chondrosarcoma are the most common subtypes. While difficult to estimate, incidence rate per 100,000 person years in the Netherlands has been reported as 0.27 for osteosarcoma, 0.16 for chondrosarcoma and 0.15 for Ewing Sarcoma based on a national retrospective registry [1]. Our review will focus on osteosarcoma. Osteosarcoma disproportionally affects children and young adults [1].

It is well recognized that the standard treatment for patients with resectable osteosarcoma is multi-agent chemotherapy cisplatin (C)-doxorubicin (D) +/- high dose methotrexate (HD-MTX) (MAP) followed by oncologic resection [2], [3]. Multiple guidelines have recommended that rare bone tumours, including osteosarcoma, be treated at specialist sarcoma centres [3]. Outcomes for patients with metastatic osteosarcoma remain poor, with outcomes largely unchanged since the introduction of cytotoxic chemotherapy over 3 decades ago [4], [5], [6]. In the recent large EURAMOS-1 trial, patients with localised osteosarcoma had a 5-year event-free survival (EFS) of 60% and 5-year overall survival (OS) of 76%. Patients with metastatic disease at presentation had an inferior 5-year EFS ad OS of 28% and 45% respectively [7]. Given patients are often diagnosed in the prime of their life, identification of novel treatments is desperately needed. From stimulating host immune response to inhibiting immune-suppressive properties of tumours, the field of cancer immunotherapy has advanced rapidly in recent decades. Strategies such as checkpoint inhibition and cellular therapies are now commonly used treatments for certain cancer types after showing significant improvements in both survival and quality of life for patients in clinical trials [8], [9]. Given the success of immunotherapy in multiple cancer types, there has been a keen interest in exploring immunotherapy in osteosarcoma.

## 2 Does the osteosarcoma micro-environment suggest role for immunotherapy?

Compared to epithelial tumours, tumour associated macrophages (TAM) compose a significant amount of the osteosarcoma immune microenvironment [10]. Depending on their polarisation, TAMs can exert different effects on tumour development [11]. M2-polarised TAMs have been described to promote tumour growth due to their roles in angiogenesis and production of immunosuppressive cytokines. On the other hand, M1-polarised TAMs produce pro-inflammatory cytokines and are considered anti-tumoural.Tissue micro-arrays developed from osteosarcoma samples at diagnosis have demonstrated patients with tumours expressing activated M1-polarised macrophages were less likely to develop metastatic disease [12]. Furthermore, higher macrophage infiltration was significantly associated with improved overall survival. Buddingh *et al.* performed genome-wide mRNA expression profiling on pre-treatment biopsies of patients who did and did not develop metastasis [13]. A significant number of genes were differentially expressed; tumours from patients who did not develop metastasis had upregulation of genes involved in macrophage function. Expression of two macrophage associated genes *CD14* and *HLA-DRA* in tumour samples were independently associated with metastasis free survival within the cohort [13]. Importantly, through comparison of expression of macrophage associated genes in tumour samples and osteosarcoma cell lines, the lack of macrophage activation genes in osteosarcoma cell lines suggests that the macrophage function signalling is occurring due to hematopoietic cells within the osteosarcoma microenvironment [13]. This work forms the basis of exploration of macrophage activation as a therapeutic strategy for patients with osteosarcoma.

## 3 Therapy directed at Macrophage activation in osteosarcoma

Mifamurtide’s active ingredient is muramyl tripeptide ethanolamine (MTP-PE), which is a synthetic derivative of muramyl dipeptide (MDP) [14]. MDP occurs in the walls of bacteria and has a role in immune potentiating activity of the cell wall. MDP has been shown both *in vitro* and *in vivo* to induce monocyte and macrophage activation. This is thought to be via nucleotide-binding oligomerization domain (Nod) 2 leading to nuclear factor-κβ activation and secretion of other downstream pro-inflammatory cytokines [14], [15]. MTP-PE is encapsulated into liposomes, and these liposomes are selectively taken-up by macrophages where they form part of the phospholipid bilayer. As this bilayer is broken down, MTP-PE is released to induce a MDP-like inflammatory response. *In vitro*, mifamurtide has been shown to have anti-tumour activity via macrophage activation, direct cytotoxicity and release of tumoricidal factors [14].

In a double-blind placebo controlled trial of adjuvant mifamurtide for dogs with localised osteosarcoma, mifamurtide showed an improved EFS [16]. Given similarities in canine and human osteosarcoma [17], this study sparked interest in exploring the role of mifamurtide in humans. Early phase trials with mifamurtide are well summarised elsewhere [14]. Given the non-overlapping mechanism and toxicity with standard chemotherapy, a phase III trial was designed to determine if the addition of mifamurtide would improve outcomes for patients (age 0–30 years) with resectable osteosarcoma [18] using two different chemotherapy backbones to also understand the role of ifosfamide. In total, 667 patients were randomised after initial diagnostic biopsy to one of four treatment arms (Table 1). Final results showed no difference in EFS or OS between patients treated with ifosfamide containing regimen and those who receive standard of care MAP. There was a statistically significant increase in OS (70% versus 78%, p = .03), but not EFS for patients treated with mifamurtide and either chemotherapy backbone. However, these data must be interpreted with caution. The trial design randomised patients at initial biopsy, even though mifamurtide was given after surgery. The groups were therefore not balanced according to response to neoadjuvant therapy. This is a key, well-studied prognostic factor for patients with osteosarcoma, as patients with > 90% necrosis post neoadjuvant therapy consistently have improved outcomes compared to those with < 90% necrosis [19], [20], [21], [22]. Imbalance in this factor may confound the results, and impact of this imbalance may be under-estimated as percent necrosis was unknown for 10% of tumours. Notably, while the 6-year OS for the total population was higher for patients who received mifamurtide, the major benefit was seen in patients in Arm B (Table 1). Arm B used a backbone which includes ifosfamide, and was not superior to MAP, and is not used in clinical practice. Finally, a significant proportion of patients (n = 94/667, 15%) could not be evaluated for survival from definitive surgery, which also weakens the final results of the study. In totality, this study provides an initial clue that immunomodulatory treatments may have a role in osteosarcoma. However, it is unclear which patients truly benefit from the addition of mifamurtide. Based on these phase III results, mifamurtide has been approved in the USA, Israel, Korea, Mexico and Taiwan [2]. While it is approved in several countries, there is no international consensus regarding the use of mifamurtide [3]. There is an ongoing phase II French Sarcome-13/OS2016 study of mifamurtide for patients with newly diagnosed osteosarcoma. This trial will randomise patients post operatively stratified by metastatic status and pathological response. However, the chemotherapy backbone for patients < 25 years of age is methotrexate-etoposide-ifosfamide [23], and for those 25–50 it is AP + A-Ifosfamide. These regimens are not widely used and may limit the generalisability of results for OS2016 [23].

**Table 1:** Summary of the Phase III Trial of Mifamurtide in Resectable Osteosarcoma [14], [18], [24]

ARM	Induction	Adjuvant / Maintenance Therapy	EFS 6 year (%)	Survival 6 year (%)	% of Patients with Huvos Grade 3 or 4 Necrosis (>90% necrosis) [25]	% of Patients with Huvos Grade 1 or 2 (<90% necrosis)	Unknown Necrosis %
A-1	Doxorubicin Cisplatin HDMTX		64	71	44.1	45.3	10.7
A-2	Doxorubicin Cisplatin HDMTX	+Mifamurtide	63	75	56.6	33.8	9.8
B-1	Doxorubicin Ifosfamide HDMTX	+ Cisplatin	58	70	47.3	43	9.8
B-2	Doxorubicin Ifosfamide HDMTX	+ Cisplatin +Mifamurtide	71	81	43.5	42.9	13.7

## 4 Role of interferon

Beyond macrophage activation, there was interest in interferon as a novel addition to standard treatment of osteosarcoma. Interferons are cytokines with a multitude of roles within the body. Type I interferons, which include interferon α and interferon β, bind to the type I interferon receptor (IFNAR) and activate downstream signalling pathways through the activation of Janus activated kinases (JAKs) tyrosine kinase 2 (TYK2) and JAK1 [26]. Interferon γ, a type II interferon, signals through the interferon γ receptor (IFNGR). Interferons can directly inhibit growth of tumour cells, or indirectly through the regulation of major histocompatibility complexes (MHCs) on antigen presenting cells and activation of macrophages and cytotoxic T cells [27].

Initially discovered for their anti-viral effects [28],interferons were first proposed to have anti-cancer properties following their ability to improve survival of mice with viral-induced leukaemias [29]. This was soon confirmed in subsequent *in vivo* studies where interferons were found to inhibit the growth of transplanted tumours of various origins in mice [30]. For the treatment of osteosarcomas in the preclinical setting, interferon α has been shown *in vitro* and *in vivo* to directly inhibit growth of human osteosarcoma cell lines and patient derived xenograft models in mice [31], [32], [33]. The Karolinska Hospital treated 102 consecutive patients with interferonα post operatively from 1971 to 1990. The metastasis free survival at 10 years was 39%; suggesting there may be a role for interferon in adjuvant treatment of osteosarcoma [34]. As part of the EURAMOS-1 trial, patients with good histological response (>90% necrosis) after at least 2 cycles of neoadjuvant MAP were randomised to adjuvant treatment with 4 additional cycles of MAP +/- maintenance pegylated interferon-α2b (IFN) weekly for weeks 30–104 [35]. A significant number of patients did not start IFN (24%, n = 86/357) and many (39%, n = 105/271) stopped early due to toxicity. This limits the interpretation of results in an intention to treat analysis. Three-year EFS for the entire population was 73%, with no difference in EFS seen for patients treated with IFN. The results of this trial highlight the importance of incorporating quality of life metrics into trials; particularly when there is already a high baseline toxicity with standard of care treatment (i.e. MAP). Given the significant patient drop out due to toxicity of interferon, any incorporation of interferon into future trials must be carefully considered.

## 5 Clues for a Future role for immunotherapy

Programmed death receptor-1 (PD-1) is an inhibitory receptor expressed on the surface of activated T cells and other immune cells [36]. When phosphorylated by the ligand of PD-1 (PD-L1), Src homology 2 domain-containing tyrosine phosphatase 2 (SHP2) is recruited to initiate signalling pathways involved in immune-suppression [37]. PD-L1 is expressed in immune cells but is also commonly upregulated in tumour cells, allowing evasion of immune surveillance. Inhibition of the PD-1/PD-L1 interaction using anti-PD-1 or anti-PD-L1 antibodies have shown clinical benefit and have been approved for use to treat multiple cancer types [38], [39].

Osteosarcoma cell lines and tumour samples have been shown to express PD-L1 [40], [41]. PD-1 and PDL-1 expression is present in both de-calcified and calcified patient tumour specimens [41], making it a feasible biomarker. No difference was seen in PD-L1 expression in primary tumours versus metastatic tumours [40]. High PD-L1 expression on initial tumour sample was associated with significantly poorer 5 year EFS (25% versus 69.4%) [40]. Studies using preclinical osteosarcoma mouse models showed reduced metastasis and prolonged survival when mice were treated with anti-PD-1 antibody or a combination containing anti-PD-1 and anti-PD-L1 antibodies [42], [43]. Thus, PD-L1 inhibition has become an attractive therapeutic target in this disease. However, despite having variably high PD-L1 expression, the overall response rate (ORR) is only 1.4% across multiple trials including 69 patients with advanced osteosarcoma (n = 1/69) (Table 2). There are ongoing studies including a phase II for single agent avelumab [44]. While there is biological rationale for checkpoint inhibition in osteosarcoma, it is clear that checkpoint inhibition alone is insufficient. Future work is needed for novel combination treatments which include checkpoint inhibitors.

**Table 2:** Summary of Recent Checkpoint Inhibitor Trials in Advanced Osteosarcoma.

	Number of Patients with Osteosarcoma	Population	% PD-L1 +	Checkpoint Inhibitor	Response Rate (Partial Response + Complete Response)	mPFS (months)	mOS (months)
Boye *et al* 2021 [45]	12	>18yo; Relapse or progression after 1L or more of systemic therapy	9% (n = 1/11)	Pembrolizumab 200 mg IV q3weeks	0	1.7	6.6
Geoerger *et al* 2020 [46]	10*	<17yo; Advanced, relapsed or refractory solid tumour	33% (n = 18/54)^	Pembrolizumab 200 mg IV q3weeks	0	NR	NR
Davis *et al* 2020 [47]	13*	<18yo; Recurrent or refractory solid tumours	22% (n = 2/9)	Nivolumab 3 mg/kg q2weeks	0 responses in solid tumours in the trial	NR	NR
Geoerger *et al* 2020 [48]	12*	<30yo; no curative standard of care options exist	83% (n = 5/6)	Atezolizumab 15 mg/kg (<18yo) or 1200 mg (>18yo) q3weeks	0	NR	NR
Tawbi *et al* 2017 [49]	22*	>12yo; metastatic or unresectable disease	NR	Pembrolizumab 200 mg IV q3weeks	5% (n = 1/22)	NR	NR

*Subgroup within a larger trial

NR = not reported for the individual subset

^For all screened patients

Exhaustion of tumour infiltrating lymphocytes (TILs) has been shown to occur over time in the tumour microenvironment [50] and depletion of TILs has been shown *in vivo* to accelerate progression of metastatic osteosarcoma in preclinical mouse models [51]. Additionally, Théoleyre and colleagues showed that human TILs isolated from biopsies of osteosarcoma patients were cytotoxic against osteosarcoma and other tumour cell lines *in vitro*, suggesting a therapeutic potential for the treatment of osteosarcoma [52]. To rationally exploit this, Zhou *et al*. treated 60 patients with metastatic osteosarcoma previously treated with chemotherapy with nivolumab q2weeks and injected individual patient derived cultured TILs from fresh tumour biopsies. The ORR by response evaluation criteria in solid tumours (RECIST) 1.1 was 36.7% (n = 22/60, 2 complete response (CR) and 20 partial response (PR)), with the median progression free (mPFS) and overall survival (mOS) 5.6 months and 13.6 months respectively for the entire population [53]. Patients with an objective response had a longer mPFS (8.9 months) and mOS (23.7 months). Higher TIL infusion numbers was associated with a response [40]. This small retrospective study suggests that there may be a role for combined approaches to immunological treatments. Given a small subset of patients had a CR, translational work is required to understand the unique tumour and tumour microenvironment characteristics that identify patients who may derive significant benefit from such a combined approach.

Cyclophosphamide is an alkylating agent used alone or in combination with other therapies to treat various cancers [54]. Low-dose cyclophosphamide was first shown *in vivo* in a carcinoma rat model, and subsequently in patients with a range of metastatic tumours to transiently reduce accumulation of immune-suppressive regulatory T cells [55]. In a mouse osteosarcoma model, cyclophosphamide showed a synergistic effect on immunostimulation when combined with an anti-PD-1 antibody [56]. Based on the immunomodulatory role of cyclophosphamide [55], the phase II PEMBROSARC study of metronomic cyclophosphamide and pembrolizumab was designed by the French Sarcoma Group [57]. Results from 15 patients over 18 years old demonstrated a best ORR of 7% (PR n = 1/15) and 33.3% (n = 5/15) of patients having stable disease as best response. Interestingly, 4 patients had tumour shrinkage with only 1 meeting RECIST 1.1 criteria for PR. However, of these patients who had available tumour (n = 3/4), none had expression of PD-L1 on tumour or immune cells, suggesting that PD-L1 may not be the correct predictive biomarker. Within this cohort, clinical benefit was short, with a 6 month non-progression rate of 13.3% and mPFS 1.4 months [57]. The first tumour response assessment at 6 weeks and the mPFS being 1.4 months suggests that cyclophosphamide may not be the correct combination treatment to pair with typically slow responding immunotherapy [58]. Rather, within a similar trial design, the cytotoxic treatment paired with immunotherapy should have a high initial response rate to prevent rapid initial disease progression to allow time for immunotherapy to work.

## 6 Beyond PD-L1/PD-1 inhibition

B7-H3 is a T cell regulator involved in cell mediated immune response through both co-activation and co-inhibition [59]. Overexpression of B7-H3 has been shown to correlate with poor OS across multiple cancer types [60]. In a study of 61 pre-treatment osteosarcoma tumour specimens found 60.7% of specimens had strong B7-H3 expression by immunohistochemistry (IHC). Higher B7-H3 expression was associated with more advanced stage, development of pulmonary metastasis and poorer overall survival [61]. Overexpression of B7-H3 correlated with reduction in numbers of CD8 + T-lymphocytes compared to tumours with low B7-H3. This suggests that B7-H3 may play an immune suppressive role which promotes aggressive behaviour in a subset of patients with osteosarcoma. After showing potent anti-tumour activity *in vitro* and *in vivo* in several B7-H3 expressing cancer types, B7-H3 targeting monoclonal antibodies (enoblituzumab) and chimeric antigen receptor (CAR)-T cell based therapies are now being tested in early phase trials [62]. A phase 1 study of enoblituzumab in pediatric patients, including those with osteosarcoma, completed May 2019 [63]. However, final results have yet to be published [64].

## 7 Cellular therapy

There has been interest in exploring cellular therapies based on preliminary results of using genetically modified T cells in other sarcomas [65]. CAR-T cell therapy involves genetic modification of T cells collected from patients to express a chimeric antigen receptor that recognises an antigen specific to the patient’s tumour. When reintroduced back to the patient, the CAR-T cells are able to specifically target tumour cells expressing the antigen [66].

Human epidermal growth factor receptor 2 (HER2) is expressed in up to 60% of osteosarcomas [67]. However, the expression level is low and HER2 monoclonal antibodies such as trastuzumab have limited effect against osteosarcoma. With the aim of overcoming this, there has been great interest in using HER2 expressing CAR-T cells for osteosarcoma. Ahmed and colleagues showed that HER2 expressing CAR-T cells were able to target and kill HER2 positive osteosarcoma cell lines *in vitro* despite low expression levels [67]. HER2 expressing CAR-T cells also caused regression of osteosarcoma xenografts in locoregional and metastatic mouse models. A phase I/II study explored escalating doses of HER2 expressing CAR T Cells to patients with HER2 positive sarcomas has been completed. Most patients had osteosarcoma (n = 16/19); best response for evaluable patients was 0% (n = 0/14) with 1 patient having stable disease. However, the challenge in osteosarcoma lies both in identification of an appropriate cancer-specific antigen [68] and whether this antigen truly is predictive of benefit from T cell therapy. One such target maybe be ganglioside GD2; a glycosylated lipid molecule present on plasma membranes of limited normal tissues [69], [70], [71]. The majority of primary and metastatic osteosarcoma have shown to express ganglioside GD2 [69], [70]. Long *et al*. showed that GD2 expressing CAR-T cells were effective at killing GD2 positive osteosarcoma cell lines *in vitro,* but had minimal anti-tumour effect against an *in vivo* osteosarcoma xenograft mouse model when administered alone [72]. Despite this, there are a number of ongoing trials exploring cellular therapy using GD2, including the phase I GD2-CAR PERSIST [73] and VEGAS [74] which have completed accrual and another phase I trial currently recruiting [75]. Results are eagerly awaited to help guide further research.

## 8 Future directions

Recent whole genome sequencing working on osteosarcoma samples has demonstrated the genomic complexity of these tumours [76]. Alterations were seen across multiple genes including *MYC*,*CCNE1*, *RAD21*, *VEGFA*, *AURKB*, *CDK4*, *TP53*, *RB1*, *PTEN*, with no clear signature or sub-grouping of patients emerging. However, it was possible to group patients by somatic copy number alteration (SCNA) and subsequently pair available therapies to SCNAs (i.e. palbociclib and CDK4 [76], [77]. However, SCNA groupings included *CDK4*, *AURKB*, *PI3K-AKT-mTOR* and *VEGF*, but did not cluster with immune-associated pathways. Furthermore, while a recent analysis of the immuno-genomic landscape of osteosarcoma was able to identify three subgroups categorised by distinct levels of immune cell infiltrate, the authors found a prevalence of multiple mechanisms of immune suppression that facilitate immune evasion and a lack of T-cell activation [78]. These studies demonstrate the complexity that is inherent amongst individual osteosarcoma patients. Future molecular profiling efforts in larger patient cohorts by The Osteosarcoma Project [79] and the ICONIC trial [80] may provide further resolution on the underlying complexity in this disease.

This frustrating complexity is the major hurdle to the stagnant survival outcomes for patients with osteosarcoma over the past 4 decades. While in general, results from immunotherapy have been disappointing with few patients responding, the question remains regarding understanding which subset of patients may have long term benefit from immunotherapy-based approaches. It may be that potentiating the immune response through a combining macrophage checkpoint inhibition (i.e. anti-CD47 inhibitor [81] with PD-L1 inhibitors or macrophage activators (i.e. mifamurtide) benefits a greater number of patients. Given their renaissance the COVID19 pandemic, mRNA vaccines may provide a rational augmentation strategy to conventional immunotherapy [82]. Given the success of immunotherapy-chemotherapy combinations in other tumour types [83], [84] another potential means of improving immune therapy a potential strategy may be rational selection of the immunotherapy partner to enable patient’s early disease control to allow for time for immunotherapy to take effect. For patients with metastatic disease, an ongoing challenge is identifying effective treatments for patients beyond first line MAP based chemotherapy. Incorporation of immunotherapy-based approaches may require a standard immunotherapy backbone in combination with patient specific targeted treatment based on tumour specific whole genome sequencing to overcome the genomic diversity within osteosarcoma. Such an iterative translational approach is required to improve outcomes in such a heterogenous patient population. In addition, there is some early evidence that the B cell infiltrates and the presence of tertiary lymphoid structures may be predictive of immune checkpoint inhibitor response in soft tissue sarcomas with several ongoing trials evaluating this association prospectively [85]. Given that tertiary lymphoid structures and elevated B cell infiltrates have recently been described in canine osteosarcoma metastases [86] (87), it would be interesting to assess if this could serve as a potential biomarker for the selection of patients who are most likely to benefit from immunotherapy-based approaches.

## 9 Conclusion

Early studies of immunotherapies in osteosarcoma have been disappointing. Some of the challenges lie in interpretation of large trials with significant patient drop out or limited follow up data. Available evidence suggests that immuno-oncology drugs alone are unlikely to significantly impact outcomes for patients with osteosarcoma. Improved outcomes may lie in rationally translational trials to target the heterogeneity amongst osteosarcoma.

## 10 Funding source

The authors acknowledge funding to the Royal Marsden/Institute of Cancer Research National Institute for Health Research Biomedical Research Centre. This report is independent research funded by the National Institute for Health Research.

## Conflicts of Interest

R.L.J. is the recipient of grants/research support from MSD, GSK. RLJ is the recipient of consultation fees from Adaptimmune, Athenex, Blueprint, Clinigen, Eisai, Epizyme, Daichii, Deciphera, Immunedesign, Lilly, Merck, Pharmamar, UptoDate. PA has a patent on glutamine + trehalose combinations to reduce mucositis (commercial product is Healios distributed by Enlivity.com and Amazon). All other authors have no conflicts of interest to declare.

### CRediT authorship contribution statement

**Alannah Smrke:** Conceptualization, Data curation, Formal analysis, Writing – original draft, Writing – review & editing. **Yuen B. Tam:** Conceptualization, Data curation, Formal analysis, Writing – review & editing. **Peter M. Anderson:** Conceptualization, Formal analysis, Writing – review & editing. **Robin L. Jones:** Conceptualization, Data curation, Formal analysis, Writing – review & editing. **Paul H. Huang:** Conceptualization, Data curation, Formal analysis, Writing – original draft, Writing – review & editing.

## Declaration of Competing Interest

The authors declare that they have no known competing financial interests or personal relationships that could have appeared to influence the work reported in this paper.
